# Totally laparoscopic total gastrectomy with Uncut Roux-en-Y for gastric cancer may improve prognosis: A propensity score matching comparative study

**DOI:** 10.3389/fonc.2022.1086966

**Published:** 2022-12-22

**Authors:** Yizhen Chen, Tao Zheng, Yifan Chen, Yuanyuan Zheng, Song Tan, Shaolin Liu, Yuhang Zhou, Xiaojun Lin, Weijie Chen, Yulong Mi, Shentao Lin, Changshun Yang, Weihua Li

**Affiliations:** ^1^ Shengli Clinical Medical College of Fujian Medical University, Fuzhou, China; ^2^ Department of Surgical Oncology, Fujian Provincial Hospital, Fuzhou, China; ^3^ Department of VIP Clinic, Fujian Provincial Hospital, Fuzhou, China

**Keywords:** gastric cancer, total gastrectomy, laparoscope, uncut Roux-en-Y, digestive tract reconstruction

## Abstract

**Background:**

Laparoscopic total gastrectomy (LTG) with Roux-en-Y (RY) is often accompanied by a series of complications. Uncut RY (URY) can effectively reduce Roux stasis syndrome (RSS) in laparoscopic distal gastrectomy. To determine whether totally LTG (TLTG) with URY for gastric cancer (GC) can replace RY in short-term and long-term prognosis.

**Methods:**

This comparative retrospective study selected GC patients from 2016 to 2022. The patients were divided into URY group and RY group. Cox multivariate proportional hazard regression analysis was used to explore the independent prognostic factors. Propensity score matching (PSM) was used to reduce bias.

**Results:**

A total of 100 GC patients met the inclusion criteria. Compared to RY group, URY group showed significant advantages in operation time and length of hospital stay. In addition, URY group can significantly reduce short-term and long-term complications, especially RSS. The 1-, 3- and 5-year progression free survival (PFS) of URY group and RY group were 90.4% *vs.* 67.8% (P=0.005), 76.6% *vs.* 52.6% (P=0.009) and 76.6% *vs.* 32.8% (P<0.001), respectively. After PSM, the advantage of URY in PFS was verified again, while there was no significant difference in overall survival (OS) between the two groups. Cox multivariate analysis suggested that lower RSS was associated with better PFS.

**Conclusions:**

TLTG with URY for GC helps control disease progression, speed up recovery and reduce short and long-term complications, especially RSS.

## 1 Introduction

According to the latest cancer statistics, gastric cancer (GC) is one of the most common cancers and the third leading cause of cancer-related deaths worldwide (1, 2). Radical surgery is the only possible cure for GC (3).

Total gastrectomy is the preferred surgical procedure for tumors located in the middle or proximal part of the stomach (4). With the popularization and application of laparoscopic technique in GC, assisted laparoscopic total gastrectomy (LTG) has gradually transformed into totally LTG (TLTG) (5, 6). The difficulty of this transformation lies in the reconstruction of the digestive tract under totally laparoscopy (7).

Roux-en-Y (RY) esophagojejunostomy (EJS) is still the most commonly used reconstruction method in total gastrectomy (8, 9). However, RY is often accompanied by a series of complications (10, 11). In addition to the improvement of survival, the quality of life (QoL) has also attracted more attention. In order to reduce the complications after total gastrectomy and improve the QoL of patients, researchers have been trying new anastomosis methods in recent decades. The uncut RY (URY) has been widely used in the reconstruction of digestive tract after distal gastrectomy (12). URY can effectively reduce Roux stasis syndrome (RSS) in laparoscopic distal gastrectomy (LDG) (13). Due to different reconstruction locations, whether TLTG with URY can reduce complications remain controversial. In addition, the existence of recanalization of jejunal input loops in TLTG is still a research hotspot (14, 15).

At present, there are few comparative studies on URY and RY under TLTG. Whether long-term complications after gastrointestinal reconstruction affect prognosis is a current research hotspot. Therefore, we retrospectively analyzed the data of GC patients to compare the short-term and long-term prognosis of TLTG with URY and RY for GC.

## 2 Materials and methods

### 2.1 Study population and grouping

**Figure 1:**
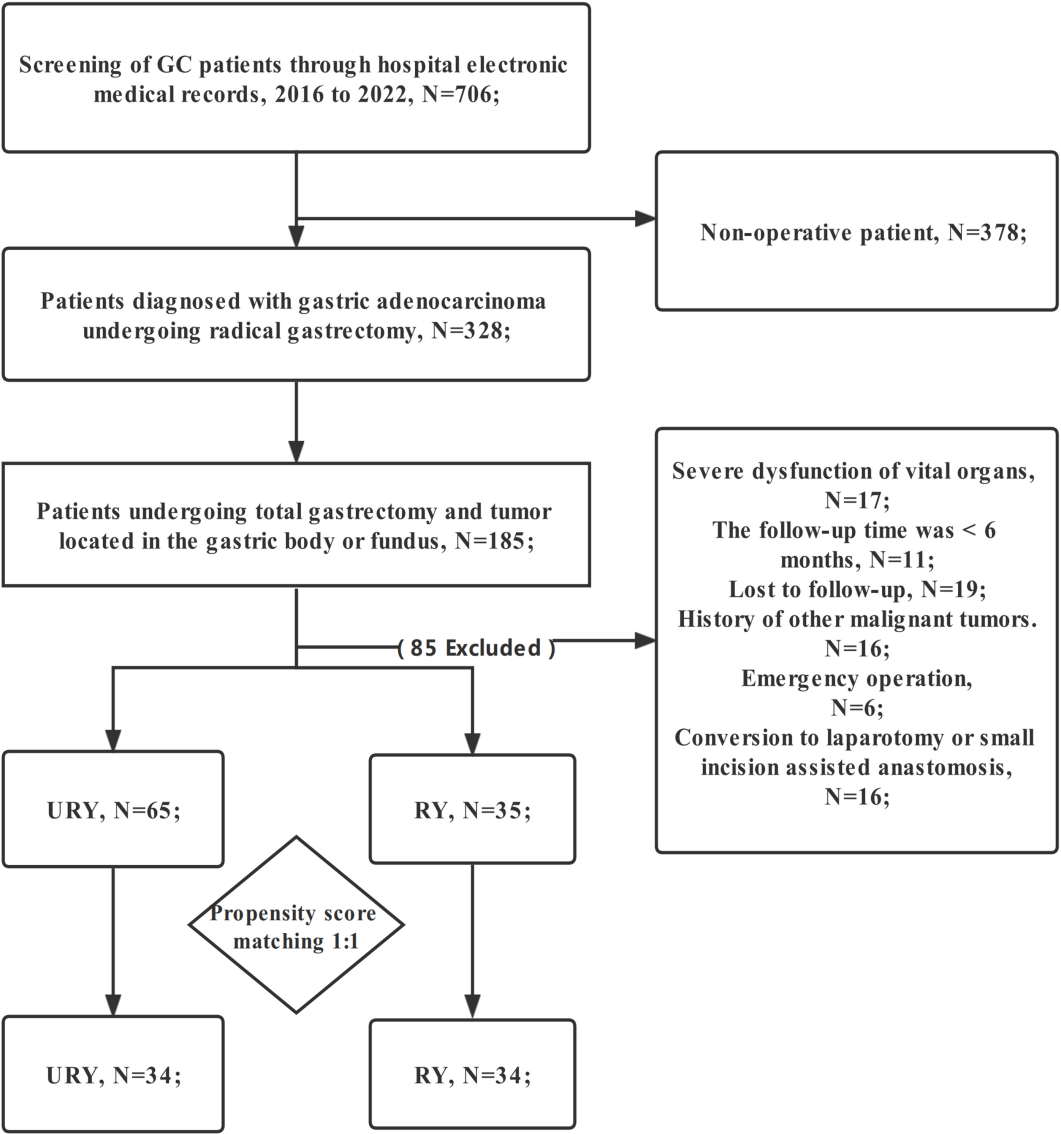
Flowchart of GC Patient Selection. GC, gastric cancer; RY, Roux-en-Y; URY, uncut RY.

The clinical data of all GC patients who underwent TLTG + EJS in Fujian Provincial Hospital from January 2016 to January 2022 were retrospectively analyzed. Eligible patients were automatically divided into two groups for comparison: RY group and URY group. The choice of anastomosis was made randomly by the chief physician during the operation. The patient did not participate in the decision-making of anastomosis mode. Patients were screened strictly according to the following criteria: (1) Gastric adenocarcinoma was confirmed by postoperative pathological examination; (2) TNM stage I-III; (3) The patient informed consent and accepted TLTG+EJS; (4) The patient has complete clinical data (including endoscopy, CT, etc.); (5) Tumor located in the gastric body or fundus. Exclusion criteria: (1) Conversion to laparotomy or small incision assisted anastomosis; (2) History of other malignant tumors; (3) Emergency operation; (4) Preoperative chemotherapy; (5) Follow-up time less than 6 months; (6) Serious organ dysfunction; (7) Lost to follow-up (**Figure 1**). Written informed consent was obtained from all GC patients. This study was approved by the Medical Ethics Committee of Fujian Provincial Hospital.

### 2.2 Surgical procedure

All patients were given general anesthesia by endotracheal intubation. The patient was positioned with the head elevated and the feet low. Both groups were performed by the same surgical team. The chief physician had more than 500 laparoscopic radical gastrectomy experience. The “five-hole method” was used to establish the endoscopic hole. Abdominal exploration showed no obvious metastasis All patients underwent standard total gastrectomy and lymph node dissection (16). The digestive tract was reconstructed as follows:

#### 2.2.1 Uncut-Roux-en-Y anastomosis

**Figure 2:**
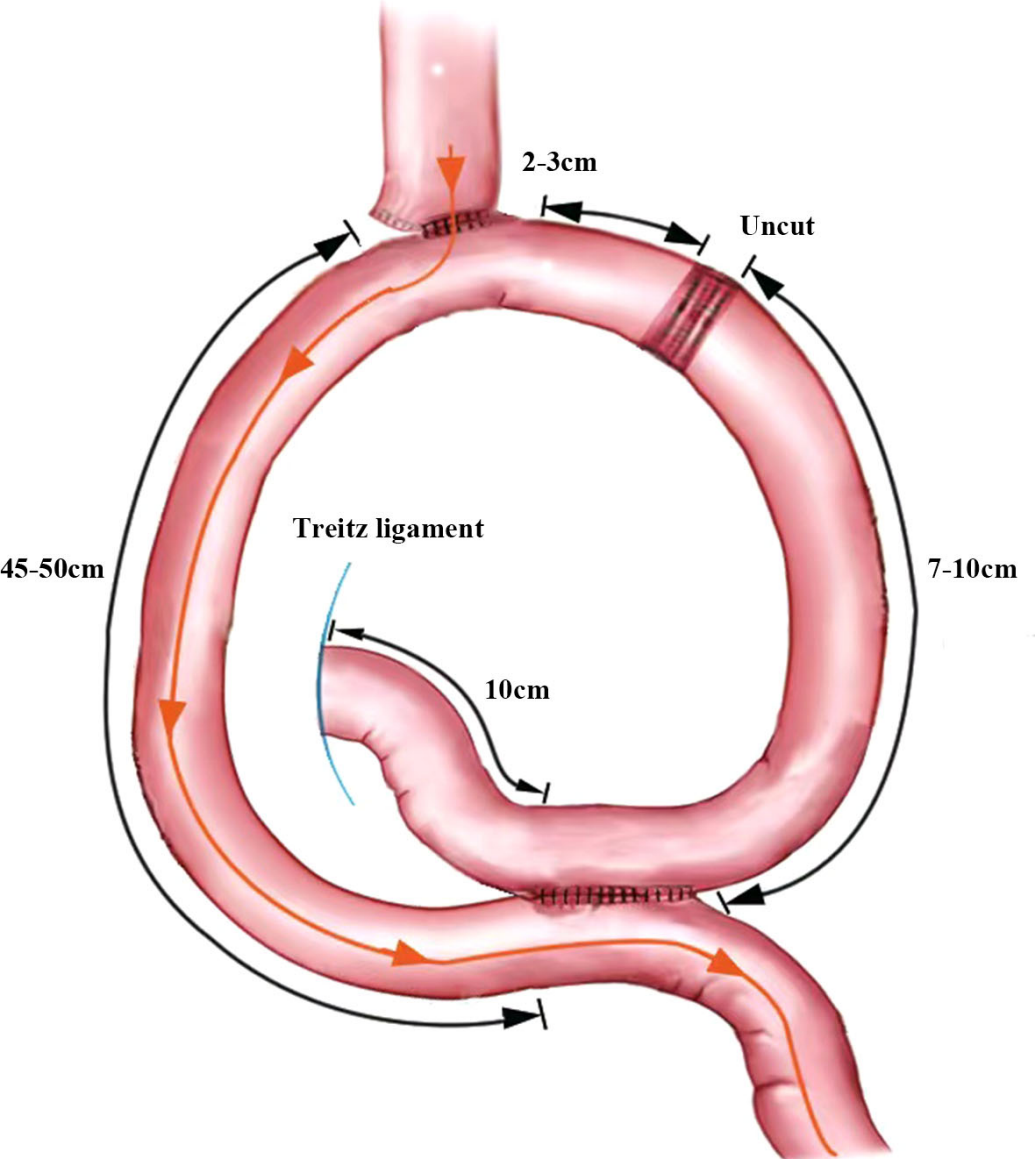
URY anastomosis diagram Orange arrows indicate the direction of food flow.

The jejunum, approximately 25 cm from the Treitz ligament, was raised to the lower esophagus, where a small incision was made on its lateral side. The two arms of the linear cutting closure are inserted through the esophageal, jejunal incision, respectively. And then the anastomosis is completed. The common opening of the esophagojejunal was finally closed with closure. A small incision was made into the distal jejunum approximately 45 cm from the esophagojejunostomy and the proximal jejunum approximately 10 cm from the Treitz ligament, respectively. A jejunal Braun anastomosis was then performed. The input loop jejunum 2-3 cm away from the esophagojejunostomy was closed with an uncut linear cutting closure (ATS45NK, Johnson & Johnson, USA). The specimen is placed into the specimen bag after completion of the URY anastomosis (**Figure 2**).

#### 2.2.2 Roux-en-Y anastomosis

The jejunum, approximately 15-20 cm from the Treitz ligament, was cut with a linear cutting closure. The distal jejunum was raised to the esophageal. After stapler insertion in the esophagus and distal jejunum, an esophagojejunostomy was performed anterior to the colon. A small incision was made in the distal jejunum 45 cm away from the esophagojejunostomy. The proximal jejunum was anastomosed to the incision. Several stitches were added around each anastomosis. The specimen was placed into the specimen bag after completion of the RY anastomosis.

### 2.3 Definitions

The primary endpoint of this study was progression free survival (PFS). Secondary endpoints were RSS and other long-term complications related to EJS. PFS was defined as the time from TLTG to confirmed death or recurrence. Overall survival (OS) was defined as the time from TLTG to the last follow-up or death. Operation time: from skin incision to abdominal closure. Intraoperative anastomosis time: from the beginning of EJS to the end of all anastomosis. Postoperative complications were divided into short-term and long-term complications. Short-term complications were graded using the Clavien-Dindo classification (17). Definition of RSS was followed as: 1. Roux-en-Y anastomosis or Uncut-Roux-en-Y anastomosis was performed to reconstruct the digestive tract; 2. Intestinal obstruction such as nausea, vomiting, and abdominal distension still occurred 3 months after surgery; 3. Imaging or gastroscopy showed food retention in the loop of Roux. And the reasons for mechanical intestinal obstruction, anastomotic stenosis, ulcers, and tumor recurrence were excluded (13, 18–20). Our postoperative feeding protocol is as follows: Patients are allowed to drink water on the first day after gastrectomy. About 1 to 3 days after removal of the gastric tube, patients are allowed to eat liquid diet, such as rice soup. The specific time of liquid and semi-liquid diet was evaluated according to the recovery of intestinal function such as postoperative ventilation. About 4 to 7 days after removal of the gastric tube, patients are allowed to add a semi-liquid diet, such as porridge.

### 2.4 Data collection and follow-up

Baseline characteristics, perioperative information and complications information were obtained from the hospital electronic medical record systems. Follow-up until October 1, 2022, to obtain the survival status and long-term complications of GC patients. The follow-up protocol was in strict accordance with the guidelines and consensus, and clinical symptoms were recorded at each review. Each GC patient returned to the hospital every three months after TLTG to confirm disease progression by endoscopy, upper gastrointestinal contrast or CT. The follow-up interval was set to 6 months if the GC patient was progression-free in the initial 2 years. The frequency of follow-up was adjusted to once a year if the progression-free status was maintained over 5 years.

### 2.5 Statistical analysis

For categorical data, patient baseline characteristics are expressed as proportions. For continuous data, mean ± standard deviation is used. The Continuity correction or Pearson’s χ^2^ is used to compare the baseline characteristics or categorical data of the two groups. Differences in continuous variables between groups are assessed using the student’s t-test. OS and PFS of the two groups were estimated using the Kaplan–Meier method and compared using log-rank test. The Cox proportional hazard multiple regression model was established. Univariate analysis was conducted firstly, and related factors (P<0.1) were included in multivariate analysis. In the multivariate analysis, factors with P<0.05 were considered as independent predictors of OS and PFS.

To simulate randomization and further address confounding factors between the two groups, a propensity score matching (PSM) analysis was used in this study. The factors with statistical difference (T stage, N stage and TNM stage) in the two groups of baselines were put into the matching variables. Then, the two groups were formed using a one-to-one nearest neighbor caliper with a width of 0.05. All statistical analyses were performed using SPSS statistical software (version 25, SPSS Inc., Chicago, IL, USA).

## 3 Results

### 3.1 Clinicopathological characteristics

From 2016 to 2022, a total of 100 GC patients met the inclusion criteria. There were 65 patients in URY group and 35 patients in RY group. The clinical characteristics of the two groups are shown in **Table 1**. The median age of patients in URY group and RY group was 66.0 and 70.0 years, respectively. The ratio of female to male in the entire cohort was 26: 74. There were significant differences in T stage, N stage and TNM stage between the two groups. All patients had no preoperative radiotherapy or chemotherapy. Most patients are ASA I-II.

**Table 1:** Baseline characteristics of the including patients.

Variables	URY	RY	P^†^
N	65	35	
Gender			
Female/Male	20/45	6/29	0.138
Age (years)			
≤60/>60	14/51	4/31	0.209
ASA			
I-II/III	44/21	28/7	0.191
Location of tumor			
Gastric fundus/Gastric body	45/20	26/9	0.595
T stage			
T1-T2/T3-T4	33/32	7/28	0.003
LN metastasis			
N0/N+	37/28	12/23	0.031
TNM			
I+II/III	45/20	14/21	0.005
Differentiation degree			
High/Medium+Low	10/55	3/32	0.513

†Continuity correction and Pearson’s x^2^ test were used to analyze the basic characteristics. ASA, American Society of Anesthesiologists.

LN, Lymph node.

### 3.2 Surgical information and postoperative complications

Both groups completed standard total gastrectomy and lymph node dissection. The perioperative conditions of TLTG for GC is shown in **Table 2**. All patients were not converted to laparotomy. The total operation time, anastomosis time, length of hospital stays and liquid food intake time in RY group were significantly longer than URY group (P=0.025, P<0.001, P<0.001 and P=0.009). The intraoperative blood loss in the RY group was also significantly more than URY group (mean 210.00 ml *vs.* 98.77 ml; P=0.011). One patient in the RY group died of complications within 30 days after surgery, whereas none in the URY group. The short-term postoperative complications in the RY group were significantly higher than URY group (22.86% *vs.* 6.15%, P=0.033).

**Table 2:** Perioperative conditions.

Variables	URY	RY	P^†^
N	65	36	
Operative time (min)	230.91 ± 43.09	252.74 ± 50.20	0.025
Intraoperative anastomosis time (min)	29.62 ± 4.16	41.23 ± 3.40	<0.001
Intraoperative blood loss (ml)	98.77 ± 58.91	210.00 ± 243.08	0.011
Length of hospital stay (days)	8.63 ± 3.37	12.11 ± 4.56	<0.001
Intake time of liquid food (days)	3.06 ± 1.00	4.40 ± 2.77	0.009
Postoperative mortality in 30 days; N (%)	0 (0.00)	1 (2.78)	0.752
Overall short-term postoperative complications; N (%)	4 (6.15)	8 (22.86)	0.033
Serious complications (Clavien III-V); N (%)	1 (1.54)	1 (2.86)	1.000
Fever; N (%)	1 (1.54)	4 (11.43)	0.092
Pneumonia; N (%)	1 (1.54)	1 (2.86)	1.000
Acute pulmonary embolism; N (%)	1 (1.54)	1 (2.86)	1.000
Other; N (%)	1 (1.54)	2 (5.71)	0.580

†Continuity correction, Pearson’s x^2^ test or student’s t-test were used to analyze the basic characteristics.

Long-term postoperative complications are shown in **Table 3**. Compared to the overall short-term complications, the difference in long-term complications between the two groups was more obvious. The overall long-term complications in RY group were significantly higher than URY group (45.71% *vs.* 10.77%, P<0.001). Among them, RY group was significantly higher than URY group in RSS and Reflux esophagitis (P=0.036 and P=0.037). During the follow-up, no recanalization of the input loop was found in the URY group.

**Table 3:** Long-term postoperative complications.

Variables	URY	RY	P^†^
N	65	35	
Overall long-term complications; N (%)	7 (10.77)	16 (45.71)	<0.001
RSS; N (%)	3 (4.62)	7 (20.00)	0.036
Reflux esophagitis; N (%)	2 (3.08)	6 (17.14)	0.037
Anastomotic stenosis; N (%)	1 (1.54)	2 (5.71)	0.580
Dumping syndrome; N (%)	1 (1.54)	3 (8.57)	0.239

†Continuity correction and Pearson’s x^2^ test were used to analyze the basic characteristics. RSS: Roux-Y stasis syndrome.

### 3.3 Survival analysis

Both groups completed R0 resection. All enrolled GC patients were reviewed strictly according to the follow-up protocol, with a median follow-up time of 27.5 months. This study uses PSM to solve the offset of the two groups of baselines. After PSM, we obtained a one-to-one matched cohort of URY and RY groups (34 GC patients per group) (**Table 4**). In the matching cohort, no significant differences in confounding factors were detected between the two groups. In addition, after PSM, compared to the RY group, the advantages of the URY group in intraoperative anastomosis time, intraoperative blood loss, length of hospital stay, short-term and long-term postoperative complications were verified again (**Table 5**).

**Table 4:** Baseline characteristics of the including patients after PSM.

Variables	URY	RY	P^†^
N	34	34	
Gender			
Female/Male	11/23	6/28	0.161
Age (years)			
≤60/>60	8/26	4/30	0.203
ASA			
I-II/III	23/11	27/7	0.272
Location of tumor			
Gastric fundus/Gastric body	23/11	25/9	0.595
T stage			
T1-T2/T3-T4	7/27	7/27	1.000
LN metastasis			
N0/N+	12/22	12/22	1.000
TNM			
I+II/III	14/20	14/20	1.000
Differentiation degree			
High/Medium+Low	3/31	3/31	1.000

†Continuity correction and Pearson’s x^2^ test were used to analyze the basic characteristics. LN:Lymph node.

**Table 5:** Operation conditions and complications after PSM.

Variables	URY	RY	P^†^
N	34	34	
Operative time (min)	241.32 ± 43.64	251.06 ± 49.94	0.395
Intraoperative anastomosis time (min)	30.59 ± 4.42	41.15 ± 3.42	<0.001
Intraoperative blood loss (ml)	108.53 ± 69.16	207.35 ± 246.22	0.030
Length of hospital stay (days)	8.62 ± 2.15	12.00 ± 4.58	<0.001
Intake time of liquid food (days)	3.12 ± 1.07	4.15 ± 2.36	0.025
Postoperative mortality in 30 days; N (%)	0 (0.00)	1 (2.94)	1.000
Overall short-term postoperative complications; N (%)	2 (5.88)	7 (20.59)	0.152
Overall long-term complications; N (%)	4 (11.76)	16 (47.06)	0.001
RSS; N (%)	3 (8.82)	7 (20.59)	0.171

†Continuity correction, Pearson’s x^2^ test or student’s t-test were used to analyze the basic characteristics.

**Figure 3:**
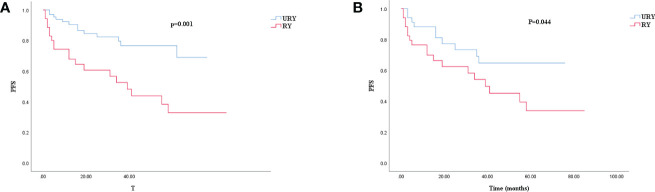
Kaplan–Meier survival curve for PFS of URY group and RY group. **(A)** was unmatched analyses and **(B)** was propensity-score-matched analyses.

Compared to the RY group, PFS of GC patients in URY group was significantly improved before and after PSM (log-rank; P=0.001, and P=0.044) (**Figures 3A, 3B**). The 1-, 3-, 5-year PFS of URY group and RY group were 90.4% *vs.* 67.8% (P=0.005), 76.6% *vs.* 52.6% (P=0.009), 76.6% *vs.* 32.8% (P<0.001), respectively. After PSM, the 1-, 3-, 5-year PFS were 88.1% *vs.* 76.5% (P=0.072), 64.7% *vs.* 54.1% (P=0.310), 64.7% *vs.* 33.8% (P=0.049), respectively. Cox multivariate analysis showed that poor PFS was independently associated with the presence of RSS [hazard ratio (HR), 4.462; 95% confidence interval (CI): 1.333-14.933; P=0.015) (**Table 6**). PFS was not related to the type of anastomosis (P=0.501).

**Table 6:** Analysis of prognostic factors associated with PFS.

Prognostic factor	n	Univariate		Multivariate	
		HR (95% CI)	P	HR (95% CI)	P
Group					
URY	65				
RY	35	3.188(1.573-6.460)	0.001	1.322(0.587-2.975)	0.501
Gender					
Female	26				
Male	74	1.546(0.593-4.027)	0.373		
Age (years)					
≤60	18				
>60	82	2.164(0.658-7.109)	0.204		
ASA					
I-II	72				
III	28	0.551(0.227-1.338)	0.188		
Location of tumor					
Gastric fundus	71				
Gastric body	29	0.454(0.175-1.181)	0.106		
T-stage					
T1+T2	40				
T3+T4	60	6.556(2.293-18.751)	<0.001	2.006(0.480-8.387)	0.340
LN metastasis					
No	49				
Yes	51	4.057(1.815-9.067)	0.001	2.857(0.803-10.165)	0.105
TNM					
I+II	59				
III	41	5.568(2.562-12.101)	<0.001	2.144(0.628-7.323)	0.223
Differentiation degree					
High	13				
Medium + Low	87	5.838(0.796-42.812)	0.083	2.294(0.283-18.590)	0.437
Overall short-term complications					
No	88				
Yes	12	2.212(0.955-5.122)	0.064	1.821(0.672-4.932)	0.238
Overall long-term complications					
No	77				
Yes	23	2.820(1.401-5.676)	0.004	2.155(0.742-6.255)	0.158
RSS					
No	90				
Yes	10	4.813(2.147-10.792)	<0.001	4.462(1.333-14.933)	0.015

HR, Hazard ratio; LN, Lymph node; RSS, Roux-Y stasis syndrome.

**Figure 4:**
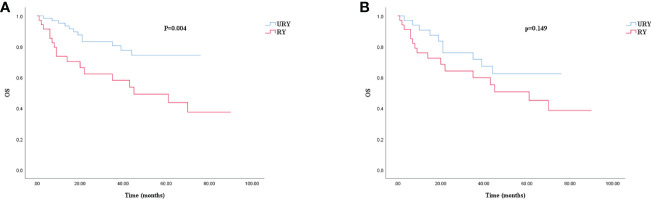
Kaplan–Meier survival curve for OS of URY group and RY group. **(A)** was unmatched analyses and **(B)** was propensity-score-matched analyses.

Compared to the RY group, the OS of GC patients in URY group was significantly improved before PSM (log-rank; P=0.004) (**Figure 4A**). However, there was no difference in OS between the two groups after PSM (log-rank; P=0.149) (**Figure 4B**). The 1-, 3-, 5-year OS of URY group and RY group were 95.3% *vs*. 73.7% (P=0.006), 80.6% *vs.* 58.2% (P=0.014), 74.4% *vs.* 49.2% (P=0.009), respectively. After PSM, the 1-, 3-, and 5-year OS were 90.8% *vs.* 76.0% (P=0.100), 71.8% *vs.* 59.9% (P=0.287), 62.5% *vs.* 50.7% (P=0.310), respectively. Cox multivariate analysis showed that poor OS was independently associated with the presence of RSS [HR, 5.538; 95% CI: 1.316-15.651; P=0.017) (**Table 7**). There was no correlation between OS and the type of anastomosis (P=0.440).

**Table 7:** Analysis of prognostic factors associated with OS.

Prognosticfactor	n	Univariate		Multivariate	
		HR (95% CI)	P	HR (95% CI)	P
Group					
URY	65				
RY	35	2.870(1.360-6.057)	0.006	1.378(0.610-3.113)	0.440
Gender					
Female	26				
Male	74	1.363(0.517-3.591)	0.531		
Age (years)					
≤60	18				
>60	82	1.946(0.588-6.433)	0.275		
ASA					
I-II	72				
III	28	0.501(0.191-1.315)	0.161		
Location of tumor					
Gastric fundus	71				
Gastric body	29	0.527(0.201-1.384)	0.194		
T-stage					
T1+T2	40				
T3+T4	60	12.352(2.933-52.020)	0.001	4.410(0.813-23.913)	0.085
LN metastasis					
No	49				
Yes	51	5.329(2.162-13.138)	<0.001	2.229(0.560-8.869)	0.255
TNM					
I+II	59				
III	41	6.838(2.910-16.065)	<0.001	2.467(0.702-8.677)	0.159
Differentiation degree					
High	13				
Medium + Low	87	5.301(0.720-39.021)	0.102		
Overall short-term complications					
No	88				
Yes	12	1.450(0.548-3.838)	0.454		
Overall long-term complications					
No	77				
Yes	23	2.301(1.094-4.842)	0.028	1.651(0.540-5.047)	0.379
RSS					
No	90				
Yes	10	3.938(1.672-9.275)	0.002	4.538(1.316-15.651)	0.017

HR, Hazard ratio; LN, Lymph node; RSS, Roux-Y stasis syndrome.

**Figure 5:**
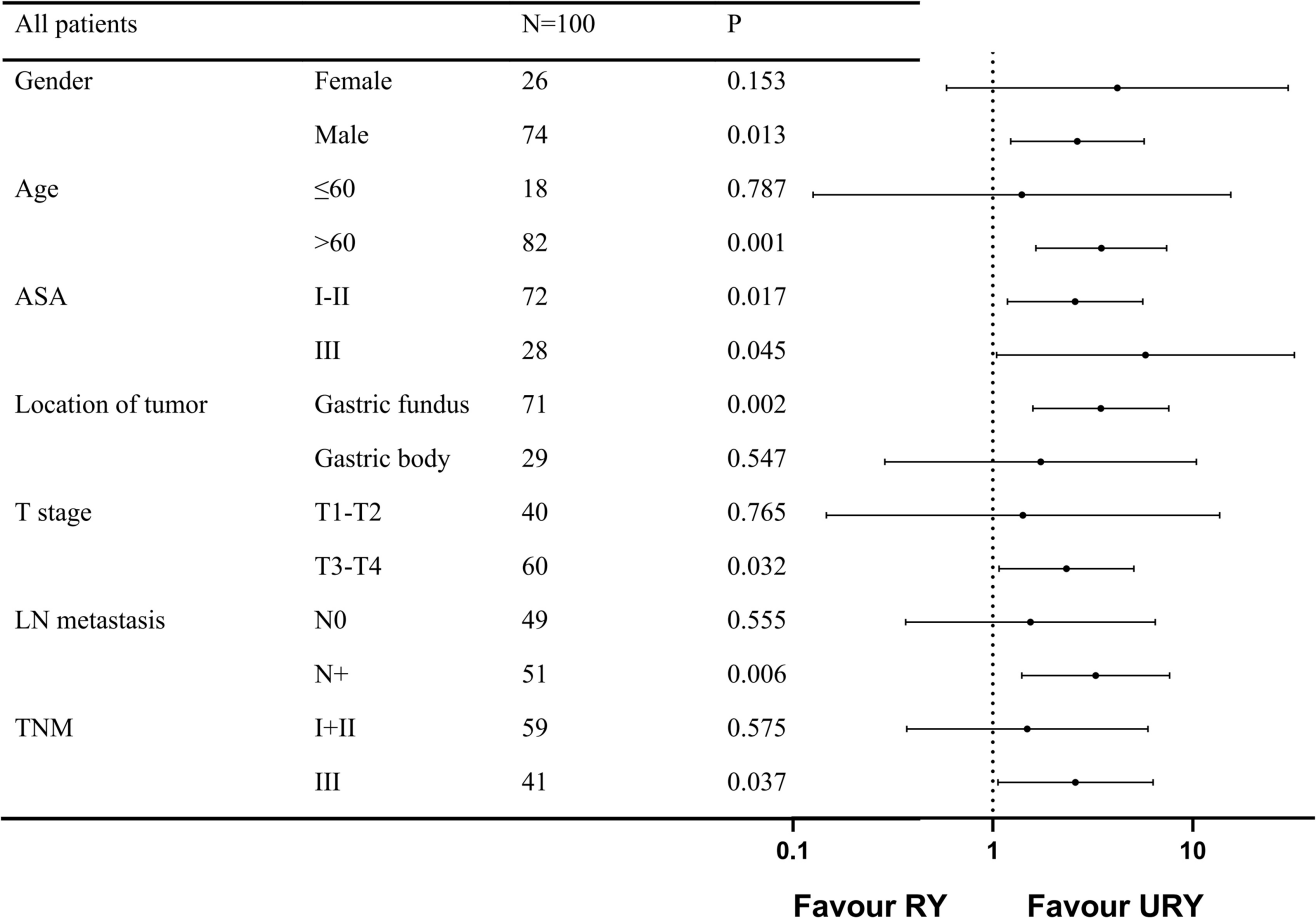
Forest plot evaluating the impact of TLTG with RY vs URY on PFS. TLTG: totally laparoscopic total gastrectomy; LN:Lymph node; RY: Roux-en-Y; URY: uncut RY.

The PFS of the two groups was different before and after PSM, subgroup analysis was used to further study the effect of anastomosis on PFS under different factors (**Figure 5**). The results showed that PFS of URY group was better than RY group in most subgroups.

## 4 Discussion

In recent years, laparoscopic surgery in the field of GC continues to progress, from laparoscopic assisted radical gastrectomy to totally laparoscopic radical gastrectomy (21, 22). However, due to the high technical requirements of digestive tract reconstruction in TLTG, which is difficult to develop and promote. It is very important to find a safe, effective and simple reconstruction method. Over the past few decades, URY has been widely used in LDG for GC (23, 24). URY can effectively reduce the incidence of RSS in LDG and improve the postoperative QoL (24–26). Whether URY is also applicable to TLTG needs further exploration. With the prolongation of postoperative survival of GC patients, the way of digestive tract reconstruction becomes the key to affect the QoL. Therefore, this study aims to explore the choice of anastomosis methods after TLTG for GC patients. To determine whether URY can replace RY in short-term and long-term prognosis.

This retrospective study mainly assessed the differences in perioperative data and prognosis between the URY and RY groups. Surprisingly, the URY group was significantly better than the RY group in PFS. PSM reconfirmed the credibility of this finding. There was no significant difference in OS after PSM. PSM simulates the randomness of prospective studies and reduces the bias caused by confounding variables. In addition, compared to RY, URY can reduce short-term and long-term complications. To the best of our knowledge, this is the first retrospective comparative study to compare whether TLTG with URY for GC has a prognostic advantage over RY. For all patients requiring total gastrectomy, we recommend URY anastomosis as the first choice without special circumstances.

In this study, only three basic clinical characteristics differed between the two groups. T stage, N stage and TNM stage are considered to be important prognostic factors in GC patients (16, 27). Clinically, TNM staging is commonly used to guide surgical treatment. The higher the stage, the worse the prognosis. The higher stage patients in the RY group were slightly more than those in the URY group. Although multivariate regression analysis of PFS or OS showed that these three confounding factors had no significant effect on prognosis in this entire cohort. But for the sake of rigor, this study used PSM to further eliminate biases.

The operation time and anastomosis time of the URY group were significantly shorter than RY group, which was predictable. Because the URY group did not need to cut off the jejunum and mesangial vessels, which simplified the surgical procedure. Of course, this can also reduce intraoperative blood loss. More intraoperative blood loss seems to be associated with the worse prognosis (18, 28). Intraoperative blood loss and transfusion should be minimized in GC surgery to improve the prognosis of patients (29). This shows the advantages of URY. From the length of hospital stay and liquid food intake time can be seen that the postoperative recovery of URY group was significantly faster than RY group. Shorter hospital stays mean less financial burden (30, 31). Of course, this is also associated with lower short-term postoperative complications. The short-term postoperative complication of the RY group was nearly 4 times the URY group. The advantage of URY in short-term postoperative complications has been repeatedly demonstrated in LDG for GC studies (25, 26). One study analyzed the life-states of 105,951 patients who underwent surgery, suggesting that patients who had complications within 30 days had a 69% reduction in median survival (32). Postoperative complications may affect postoperative treatment and long-term prognosis in certain conditions (33, 34). This may explain the poor PFS in the RY group. Therefore, the improvement of surgical quality should be aimed at preventing postoperative complications. URY has fewer postoperative complications and higher safety. URY should be actively recommended from this perspective. Of course, the effect of URY on long-term prognosis needs further study.

Postoperative QoL has always been the focus of total gastrectomy (35). Long term postoperative complications often occur after RY, which is very troublesome for postoperative treatment and QoL of patients. In LDG for GC, URY reduces RSS compared to RY (13, 19). URY can maintain the continuity of jejunum without changing the intestinal starting potential and affecting the intestinal peristalsis potential conduction (13, 36). In addition, URY has less physiological effects on the intestine, which can ensure the blood perfusion of the anastomosis and reduce the rate of anastomotic stenosis. This study also demonstrated that the overall long-term complication rate and RSS in the RY group were 2 and 4 times higher than URY group (P<0.001 and P=0.036). We also found that the incidence of RSS in RY group of this study was similar to the distal gastrectomy with RY group (11.9% - 26.4%) (23). It can be seen that the jejunum and mesentery of patients in RY group were cut off, which increased the difficulty of operation and prolonged the operation time. Eventually increased postoperative complications, which is not conducive to patient recovery. Therefore, URY anastomosis is more in line with the concept of rapid rehabilitation (37).

It is worth noting that although cox multivariate analysis suggested that PFS and OS were not related to the anastomosis. Both before and after PSM proved that URY can improve PFS compared to RY. A retrospective study of LTG for GC in 2021 compared the two anastomosis methods of RY (41 patients) and URY (45 patients) (38). The results did not find that URY could improve PFS. It may be that only 2 cases and 3 cases of RSS occurred in the URY and RY groups, respectively. We believe that the URY group improves PFS by reducing RSS, which is also confirmed by cox multivariate analysis. This is also the first time that lower RSS has been found to be associated with better PFS. There may be the following explanations for URY improving PFS by reducing RSS: ① URY can reduce short-term and long-term complications. Long-term complications such as RSS disrupt the normal treatment plan of patients after surgery, who may require postoperative adjuvant chemotherapy. There may be more patients in the URY group who can receive postoperative adjuvant therapy on time. ② The nutritional status of patients after surgery may affect the long-term prognosis of patients (39, 40). Patients in the URY group may have better nutritional status, which has been demonstrated in LDG and LTG for GC (19, 38). ③ Not cutting off the jejunum can maximize the protection of intestinal permeability. Intestinal permeability reflects intestinal mucosal barrier function (41, 42). Certain intestinal floras can also affect the treatment of tumors (43). URY may reduce tumor progression by regulating intestinal flora or improving intestinal stability. This requires further research in the future.

However, the current research has some limitations. First, this is a retrospective single-center study. There were some differences in clinical baseline characteristics between the two groups. Although PSM and cox multivariate analysis solved the bias that may be caused by clinical baseline imbalance, PSM reduced the sample size. Second, postoperative adjuvant chemotherapy information of GC patients was not collected. This is limited to retrospective studies. Finally, nutritional status was not investigated in this study. In addition to the fact that the primary and secondary endpoints of this study did not address nutritional status, they were associated with deficiencies in retrospective studies. These deficiencies will be addressed in the future through multicenter retrospective studies or prospective randomized controlled studies.

## 5 Conclusion

The results of this study are positive. TLTG with URY anastomosis technique is simple to operate. Compared to RY, URY can reduce intraoperative blood loss and operation time. URY promotes early postoperative recovery and reduces short-term and long-term postoperative complications. It can be said that URY is helpful to improve the QoL of patients after operation. In addition, URY can prolong PFS by reducing RSS,which requires further study of mechanisms. Of course, the application of URY anastomosis needs to be further validated in prospective clinical trials to guide clinical practice and application.

## Data availability statement

The raw data supporting the conclusions of this article will be made available by the authors, without undue reservation.

## Ethics statement

The studies involving human participants were reviewed and approved by The medical ethics committee of the Fujian Provincial Hospital. Written informed consent for participation was not required for this study in accordance with the national legislation and the institutional requirements.

## Author contributions

All author contributed to the article and approved the submitted version. YZ-C, TZ, YF-C, YY-Z: material preparation, search and data collection. ST, SL-L, XJ-L and YH-Z: figure preparation. YZ-C, WJ-C, and YL-M: write original draft. ST-L, CS-Y and WH-L: supervision and conceptualization. WH-L: modify the draft.
