# Corrigendum: Totally laparoscopic total gastrectomy with Uncut Roux-en-Y for gastric cancer may improve prognosis: a propensity score matching comparative study

**DOI:** 10.3389/fonc.2025.1620474

**Published:** 2025-05-26

**Authors:** Yizhen Chen, Tao Zheng, Yifan Chen, Yuanyuan Zheng, Song Tan, Shaolin Liu, Yuhang Zhou, Xiaojun Lin, Weijie Chen, Yulong Mi, Shentao Lin, Changshun Yang, Weihua Li

**Affiliations:** ^1^ Shengli Clinical Medical College of Fujian Medical University, Fuzhou, China; ^2^ Department of Surgical Oncology, Fujian Provincial Hospital, Fuzhou, China; ^3^ Department of VIP Clinic, Fujian Provincial Hospital, Fuzhou, China

**Keywords:** gastric cancer, total gastrectomy, laparoscope, uncut Roux-en-Y, digestive tract reconstruction

In the published article, there was an error in affiliation(s) “Weihua Li^1,2*^”. Instead of “Weihua Li^2*^”, it should be “Weihua Li^1,2*^”.

In the published article, there was an error regarding the affiliation(s) for “Weihua Li”. As well as having affiliation(s) “2”, they should also have “1”.

The authors apologize for this error and state that this does not change the scientific conclusions of the article in any way. The original article has been updated.

